# Primary Brainstem Hemorrhage: A Review of Prognostic Factors and Surgical Management

**DOI:** 10.3389/fneur.2021.727962

**Published:** 2021-09-10

**Authors:** Danyang Chen, Yingxin Tang, Hao Nie, Ping Zhang, Wenzhi Wang, Qiang Dong, Guofeng Wu, Mengzhou Xue, Yuping Tang, Wenjie Liu, Chao Pan, Zhouping Tang

**Affiliations:** ^1^Department of Neurology, Tongji Hospital, Tongji Medical College, Huazhong University of Science and Technology, Wuhan, China; ^2^Department of Geriatrics, Tongji Hospital, Tongji Medical College, Huazhong University of Science and Technology, Wuhan, China; ^3^Department of Neuroepidemiology, Beijing Neurosurgical Institute, Capital Medical University, Beijing, China; ^4^Department of Neurology, Huashan Hospital, Fudan University, Shanghai, China; ^5^Department of Emergency, Affiliated Hospital of Guizhou Medical University, Guiyang, China; ^6^The Department of Cerebrovascular Diseases, The Second Affiliated Hospital of Zhengzhou University, Zhengzhou, China; ^7^Beijing WanTeFu Medical Apparatus Co., Ltd., Beijing, China

**Keywords:** primary brainstem hemorrhage, prognostic factors, scoring system, surgical management, surgical options

## Abstract

Primary brainstem hemorrhage (PBSH) is the most fatal subtype of intracerebral hemorrhage and is invariably associated with poor prognosis. Several prognostic factors are involved, of which the two most predominant and consistent are the initial level of consciousness and hemorrhage size. Other predictors, such as age, hyperthermia, and hydrocephalus, are generally not dependable indicators for making prognoses. Scoring systems have now been developed that can predict mortality and functional outcomes in patients suffering from PBSH, which can thus guide treatment decision-making. A novel grading scale, entitled “the new primary pontine hemorrhage (PPH) score,” represents the latest approach in scoring systems. In this system, patients with a score of 2–3 points appear to benefit from surgical management, although this claim requires further verification. The four main surgical options for the treatment of PBSH are craniotomy, stereotactic hematoma puncture and drainage, endoscopic hematoma removal, and external ventricular drainage. Nevertheless, the management of PBSH still primarily involves conservative treatment methods and surgery is generally not recommended, according to current practice. However, the ongoing clinical trial, entitled Safety and Efficacy of Surgical Treatment in Severe Primary Pontine Hemorrhage Evacuation (STIPE), should provide additional evidence to support the surgical treatment of PBSH. Therefore, we advocate the update of epidemiological data and re-evaluation of PBSH treatment in a contemporary context.

## Introduction

Primary brainstem hemorrhage (PBSH) is a type of spontaneous brainstem hemorrhage that is particularly relevant to chronic hypertension but is not associated with definite or objective lesions such as cavernomas and arteriovenous malformations. PBSH is the most fatal subtype of intracerebral hemorrhage (ICH) and invariably has a bleak prognosis (1–3). It has the clinical characteristics of sudden onset, rapid evolution, and high morbidity and mortality (4, 5). Multiple studies have investigated the correlation between the prognosis of PBSH and its clinical features, neuroradiological presentation and neurophysiological properties (6–8). The identification of prognostic factors contributes to the development of a specific scoring system for PBSH, and a fast and accurate prognostic assessment in the emergency room plays a key role in the selection of reasonable therapeutic strategies (9). The new primary pontine hemorrhage (PPH) score represents the very latest approach in scoring systems, which will be explained below (3). It is suggested to spare medical resources for patients with a maximum score (3). However, the availability of the new PPH score for determining the surgical indications needs to be further investigated.

Actually, PBSH is currently mainly subjected to conservative treatment, and the efficacy of surgical procedures such as hematoma clearance remains questionable (4, 10–13). However, surgical interventions promise to become attractive options to manage PBSH with growing knowledge of safe entry zones into the brainstem and advances in new technologies as well as equipment in the fields of neuroimaging, microsurgery, neuronavigation, neuroendoscopy, intraoperative monitoring, and neurological rehabilitation. In this review, we aimed to analyze the identification of prognostic factors and scoring systems in PBSH and to discuss the current status and future prospects of controversial surgical management. Specifically, because PPH accounts for the vast majority (60–80%) of PBSH (5), both of these terms are used in our review, depending on the actual situation.

## Epidemiology

According to different localization of bleeding, ICH falls into two types-supratentorial and infratentorial ICH. Supratentorial ICH mainly involves basal ganglion and spontaneous infratentorial hemorrhage consists primarily of spontaneous cerebellar ICH and PBSH. PBSH occurs most frequently in the region of pontine, constituting 6 to 10% of ICH with an incidence of about 2 to 4 in 100,000 people per year and a mortality rate varying between 30 and 90% in different reports (4, 6, 14–16). PBSH occurs most often in patients aged 40 to 60, showing trends toward younger age compared with supratentorial and cerebellar ICH (17, 18). The incidence is higher in men than in women, probably because of personal living habits and health conditions prior to their illness. Hypertension is the most important risk factor of PBSH and other relative factors include anticoagulation therapy, amyloid angiopathy, etc. (16).

## Prognostic Factors

Researches, that carried out multivariate logistic regression analysis to identify independent predictors for PBSH, were shown in Table 1.

**Table 1:** Researches that used multivariate logistic regression analysis to identify independent predictors for PBSH.

**Functional outcome**								
**Author**	**Year**	**Design**	**Sample size**	**N death (duration to death)**	**N good functional outcomes**	**Follow-up**	**Independent predictors for mortality**	**Independent predictors for functional outcomes**
Dziewas et al. (14)	2003	R	39	27 (mean 16 days)	6 (mRS, 0–2)	2–8 years	Coma on admission; Hemorrhage localization; Hemorrhage size	NA
Jung et al. (19)	2007	R	35	13 (in-hospital)	12 (subjective)	Mean 13.9 months	GCS score	NA
Jang et al. (10)	2011	R	281	110 (30 days)	27 (mRS, 0–3)	90 days	Coma on admission; Dilated pupils; Respiration; Blood pressure; Hydrocephalus; Treatment modality	Coma on admission; Motor function; History of hypertension or diabetes mellitus; Eye movement; Hemorrhage size; Ventricular hemorrhage; Ventricle size
Matsukawa et al. (20)	2015	R	118	66 (follow-up period)	NA	Median 51 days	GCS score; Hyperthermia; Hemorrhage size; Hematoma extension	NA
Ye et al. (21)	2015	P	76	3 (30 days)	NA	NA	Coma on admission; Hemorrhage size; Hemorrhage localization Hemorrhage localization	NA
				56 (3 years)	NA	NA	Coma on admission; Hemorrhage size	NA
Meguro et al. (9)	2015	R	101	59 (30 days)	NA	NA	GCS score; Pupillary light reflex; Blood glucose	NA
Morotti et al. (1)	2016	R	49	30 (30 days)	NA	NA	Age; GCS score; Hemorrhage size	NA
				28 (in-hospital)	NA	NA	GCS score	NA
Huang et al. (3)	2017	R	171	68 (30 days)	74 (mRS, 0–3)	90 days	GCS score; Hemorrhage size	NA
		P	98	33 (30 days)	50 (mRS, 0–3)	90 days	NA	NA
Fan et al. (8)	2018	R	225	7 (90 days)	113 (GOS≥4)	90 days	NA	NLR; PLR; ABG; NLR-PLR-ABG
Ding et al. (22)	2020	R	136	7 (in-hospital)	30 (mRS, 0–3)	30 days	NA	Hemorrhage size; GCS score; Age; Tracheostomy
Chen et al. (7)	2021	P	31	19 (30 days)	NA	90 days	(delta + theta)/(alpha + beta) ratio (DTABR)	NA

*N, number; R, retrospective; P, prospective; NA, not available; NLR, neutrophil-to-lymphocyte ratio; PLR, platelet-to-lymphocyte ratio; ABG, admission blood glucose level; GCS, Glasgow Coma Scale; GOS, Glasgow Outcome Scale; mRS, modified Rankin Scale*.

### Demographic Factors

The incidence of ICH continues to increase as people age (4, 23). Age plays a vital prognostic role in ICH and is an important part of the ICH score (23, 24). Furthermore, previous studies showed that ICH appears to be more common in men, while women show better survival (23, 25). However, whether age or sex affects patients with PBSH remains an unresolved problem. Patient age was found to independently affect 30-day mortality or functional outcomes by Morotti et al. and Ding et al. by multivariate logistic regression analysis (1, 22). Intriguingly, no study has demonstrated that sex is a predictor of the outcomes of PBSH.

### Clinical Presentations

Coma is one of the typical symptoms of PBSH. In previous studies, depressed and poor initial levels of consciousness were usually described as “coma on admission” or measured by different GCS score critical thresholds in the range of <4 to ≤ 9 (3, 9, 16, 20, 21). In agreement, both of these descriptions could independently and reliably predict death and an unfavorable functional outcome of PBSH (6). According to Table 1, the initial level of consciousness has been identified as an independent predictor in 9 different studies, which presents the most consistent and influential predictor for PBSH. The initial level of consciousness is also simple to judge and has the potential to be part of a future PBSH-related scoring system.

Central hyperthermia is a complication after PBSH that is characterized by a core temperature of ≥39°C and is unresponsive to conventional antipyretic treatments due to an unchanged thermoregulatory setpoint (26–28). Central hyperthermia was proven to be independently related to death in PPH by Matsukawa et al., but was not identified as an independent predictive factor of 30-day outcomes after PBSH in another study (20, 22). Although central hyperthermia associated with PBSH is supposed to be injurious to patients, it remains unknown whether a positive pursuit of a normal body temperature contributes to a more favorable clinical prognosis in the absence of evidence. Therefore, future studies of patients suffering from PBSH-related central hyperthermia are absolutely essential to reveal its mechanism and preventive and treatment measures.

Additionally, patients with severe PBSH present with a high risk of neurological complications and in desperate need of measures to protect the airway, especially within the acute phase. A study revealed that early tracheostomy (≤ 7 days after admission) was significantly associated with a favorable 30-day functional outcome (prognostic benefits) and was also able to reduce the length of hospitalization and intensive care unit stay (financial benefits) (22). However, there are potential risks that should not be neglected when performing a tracheostomy, such as skin breakdown, tracheomalacia and so on (29).

In addition, other factors, such as tachycardia (>110 beat/min), absence of a pupillary light reflex, the necessity for mechanical ventilation, and pupillary abnormalities, systolic blood pressure <100 mmHg, intact motor function, and a history of diabetes mellitus, have also been identified to significantly affect death or functional outcomes after PBSH (9, 16, 28, 30).

### Laboratory Evaluation

In a retrospective study enrolling 225 patients with PBSH, Fan et al. found that elevated platelet-to-lymphocyte ratio, neutrophil-to-lymphocyte ratio, and admission blood glucose level were independently correlated with unfavorable 90-day functional outcomes of PBSH, with critical thresholds defined as 59.3, 6.65, and 7.81 mmol/L, respectively (8). Moreover, a combination of the aforementioned three factors showed a better predictive value than a single factor (8). In another study, plasma glucose with a threshold value ≥180 mmol/L (10 mmol/L) was identified to independently predict 30-day mortality in patients with PPH (9). Hyperglycemia reflects a stress-response level in the setting of the acute phase of PBSH, which could result in heightened susceptibility to complications connected with hospitalization and ultimately lead to unfavorable outcomes (31, 32).

### Radiological Evaluation

MRI findings are less relevant to prognosis after PBSH and only certain case report-level evidences have examined the potential relationship between functional prognosis after PBSH and findings derived from diffusion tensor imaging (DTI) and diffusion tensor tractography (DTT) (33–35). Instead, CT scanning is routinely deemed the method of preference for assessing PBSH owing to its general accessibility and rapid availability. Moreover, some CT findings closely correlate with prognosis in PBSH.

***1) Hemorrhage size.*** In view of the small size of the brainstem, the average hemorrhage size of PBSH is less than that of supratentorial hemorrhage but might be more fatal (36). Hemorrhage size is a reliable and significant independent prognostic factor for PBSH, of which the threshold values are found to fluctuate between 4–5 ml and 20–31.5 mm for hemorrhage volume and transverse diameter, respectively (6, 37). Hemorrhage size is another most important predictor besides the initial level of consciousness, which has been demonstrated as an independent predictor in 7 different studies in Table 1. Given the importance of hemorrhage size, inaccurate data might exert a certain adverse influence on the judgment of the prognosis. Therefore, it is probably pertinent to discuss studies on the measurement of hemorrhage volume. Computer-assisted volumetric analysis and 3D slices are typically viewed as the “gold standard” to measure hemorrhage volume (38, 39). However, the calculation process is time-consuming and tedious, hindering their clinical application. Among the formula methods, 1/2ABC is the most common and convenient way to calculate hemorrhage volume of ICH in clinical work. Nevertheless, as it may underestimate or overestimate the volume of irregularly shaped hemorrhage and small hemorrhage in the brainstem, some researchers began to question its accuracy (38, 40, 41). Through a review of 147 CT results of patients with infratentorial hemorrhage, Yang et al. found that 2/3SH was more accurate than 1/2ABC for the volume calculation of brainstem hemorrhage and irregular hemorrhage (42). As for formula 2/3SH, S represents the area of largest axial hemorrhagic slice and H represents the height of hematoma which is derived from the number of slices times the slice thickness. Overall, a more precise and simple method to measure brainstem hemorrhage size remains to be developed.

***2) Hemorrhage classification, localization, extension and hydrocephalus.*** There is currently no unified classification for PBSH (5). PBSH can be divided into three subtypes of medullary hemorrhage, pontine hemorrhage, and midbrain hemorrhage in clinical practice (43). Among them, pontine hemorrhage is the most frequent type, and isolated medullary and midbrain hemorrhages have a lower incidence (44). The medullary type may lead to ataxic respiration and cause rapid death (45). In addition, based on the axial CT findings of the exact anatomical location and spread direction, various sorts of classifications have been established (4). All of the studies are consistent with the view that unilateral tegmental hemorrhage is related to a good outcome, while massive hemorrhage (located in bilateral basal and anterior segments) is closely associated with the most unfavorable outcomes (6, 10, 14, 16, 20, 21, 26–46). For patients with neuroradiological results that fall between the two, it is difficult to predict the survival outcome according to CT findings alone (4). Intraventricular extension is an important predictive factor in ICH but is not an independent determinant of early death for PBSH patients (6, 24, 47). Jang et al. ascribed this phenomenon to the active use of external ventricular drainage (EVD), which could be conducive to the reduction of mortality and short-term prognosis in ICH (10, 48). Moreover, hemorrhage vertically extending from the pontine to the midbrain and/or thalamus could predict adverse outcomes (20). According to a systematic review, 30.3% of patients developed hydrocephalus after PPH (6). However, only one study has identified it as an independent prognostic factor of mortality for PBSH (6, 10).

***3) Hemorrhage expansion.*** In recent years, hematoma expansion has attracted wide attention in clinical practice and has been identified to independently predict mortality and functional prognosis in patients with ICH (49, 50). Hematoma shape-related signs, such as the CTA spot sign and some non-contrast computed tomography markers, have been demonstrated to be potential markers for screening out patients at high risk of hematoma expansion among ICH patients (51–53). Nevertheless, few studies have focused on hematoma expansion and relevant signs in PBSH. Even so, due to the small size but vital role of the brainstem, hematoma expansion at this site was presumed to wreak havoc on survival and prognosis. Therefore, it is an indicator to which we should attach importance in patients with PBSH. Hematoma expansion and CTA spot signs also exist in patients with PPH. In this retrospective analysis of forty-nine PPH cases, Andrea et al. found that the spot sign showed good accuracy for the prediction of in-hospital mortality (61%) and 30-day mortality (57%) but was not an independent predictor (1). In addition, the presence of spot signs was not significantly associated with hematoma expansion rates (1). However, the lack of statistical significance is ascribed to a deficient number of cases, and a clear association between spot signs and hematoma expansion rates remains uncertain.

Different from the hemorrhage size and localization, hematoma expansion has a characteristic of preventability to a certain extent. Virtual measures to restrict hematoma expansion seem beneficial to PBSH patients due to their function in reducing the ultimate hemorrhage size. Patients with PBSH are often in an urgent and high-risk state, and only a routine CT scan could be acquired. Moreover, because CTA is not available routinely in many emergency departments, the application of spot signs to predict early hematoma expansion is subject to certain restrictions (54). Under such circumstances, NCCT markers, such as the island sign (55), satellite sign (56), black hole sign (57), and blend sign (58), seem to have a clear advantage. However, as patients with PBSH are excluded from almost all relevant studies, the application value of these markers in PBSH remains unclear and needs further verification. Furthermore, the definite correlation on between hematoma expansion and PBSH and the exact mechanisms of hematoma expansion remain to be clarified. Future studies with large sample sizes are also needed to determine whether there are differences in the incidence of hematoma expansion between supratentorial hemorrhage and PBSH.

### Electrophysiological Evaluation

Although neuromonitoring is generally deemed a predictive tool for functional recovery in stroke patients, few articles have focused on the same topic in patients with PBSH (59, 60). In an analysis of 31 consecutive comatose patients with acute severe brainstem hemorrhage, Chen et al. found that a quantitative electroencephalography parameter [i.e., (delta + theta)/(alpha + beta) ratio, DTABR] could independently predict 90-day mortality, whereas no transcranial Doppler (TCD) variables showed prognostic value (7). However, that study only focused on mortality and did not attach importance to the correlation between neurophysiological parameters and functional recovery. An abnormal brainstem auditory evoked potentials (BAEPs) may predict hearing loss in PBSH as well as a poor prognosis (61). Furthermore, Seong et al. confirmed that using somatosensory evoked potentials (SEPs) and motor evoked potentials (MEPs) in combination was a reliable predictor for functional recovery in PBSH patients (62). In summary, the potential of neurophysiological parameters for predicting functional recovery still needs to be fully tapped in patients with PBSH, who often have a tendency toward severe disability.

### Scoring System

A scoring system plays an important role in the risk stratification of patients with brainstem hemorrhage, which also contributes to a consensus on their management (3, 9, 36, 63). Therefore, we discuss the development and present status of scoring systems for brainstem hemorrhage in detail.

The ICH score and its modified version are reliable and convenient and have been extensively used to predict mortality and functional recovery in ICH (24, 64). Subsequently, Del Brutto et al. revealed that both the original and modified ICH scores proved accurate for predicting the risk of 30-day mortality in PPH (63). Nevertheless, there are still some concerns. First, in the cohort used for the development of the original ICH, less than one-tenth (15 of 152, 9.87%) of all subjects were diagnosed with brainstem hemorrhage (24). Second, in light of its content, the original and modified ICH scores lead to infratentorial hemorrhage being regarded as an independent predictor of a poor outcome. Third, the cut-off value of hemorrhage size and GCS score should be different in the scoring systems for ICH and PPH. Last, a comparative study conducted by Huang et al. revealed that the original ICH score lacked discrimination and ought to be revised specifically for PPH (36). Taken together, the original and modified ICH scores may not apply well to PPH.

To solve this problem, Meguro et al. proposed the first specific grading scale (entitled the PPH score) for predicting 30-day mortality of PPH and validated it in a retrospective review of a cohort of 101 consecutive patients with PPH (9). However, the study had several flaws. The researchers did not carry out external validation and did not take into account early do not resuscitate orders (DNRs). As demonstrated by Zahuranec et al., an illusion of model accuracy may be generated when DNRs are ignored (65). Consequently, Huang et al. established and validated a new PPH score for predicting short-term outcome (30-day mortality) and long-term outcome (90-day functional prognosis) in PPH patients and demonstrated that it had a higher discrimination (area under the curve for 30-day mortality was 0.902 and that for 90-day good outcome was 0.927) and calibration than the original ICH score and the PPH score in their study cohort (3). This is the largest study with the best evidence for scoring systems to date, including a total of 269 cases (171 cases as the training set for scale development and the other 98 cases as the prediction set for external validation) (3). The detailed grading standards of these two scoring systems are shown in Table 2. Significantly, variables in the new PPH score are precisely the two most influential predictors we proposed above.

**Table 2:** Grading standards of the PPH score and the new PPH score.

**The PPH score (9)**			**The new PPH score (3)**		
**Variables**	**Range**	**Points**	**Variables**	**Range**	**Points**
GCS score	≤ 6	1	GCS score	3–4	2
	>6	0		5–7	1
Pupillary light reflex	Absence	1		8–15	0
	Presence	0	Hemorrhage volume	>10 ml	2
Blood glucose	≥180 mg/dL	1		5–10 mL	1
	<180 mg/dL	0		<5 ml	0
**Reference values**			**Reference values**		
Scores	30-day mortality rates (%)		Scores	30-day mortality rates (%)	
0	7.7		0	2.7	
1	33.3		1	31.6	
2	78.9		2	42.7	
3	100		3	81.8	
			4	100	

In terms of registered clinical studies, an ongoing trial based on the application of radiomics methods, entitled “a new prognostic scoring system for patients with primary pontine hemorrhage: medical records-based study” (URL: http://www.chictr.org.cn. Unique identifier: ChiCTR2100042705) aims to construct a new grading scale for PPH to determine the prognosis and guide therapeutic decisions.

The next step for research in scoring systems will focus on the question whether the existing system is applicable to determine the surgical indications, thereby stratifying patients and guiding treatment decision-making.

## Surgical Management

### Guidelines

Chinese researchers developed and issued the first guideline for brainstem hemorrhage in 2020 (5). However, there are no definite specifications focusing exclusively on the diagnosis and treatment of PBSH in widely recognized guidelines issued by the American Heart Association/American Stroke Association (AHA/ASA) and European Stroke Organization (66, 67). The AHA/ASA guidelines are explicitly against surgical interventions for brainstem hematomas (66). Moreover, conservative treatment of PBSH is widely accepted, whereas surgical management remains questionable because the complex anatomical structures and critical functions of the brainstem have potential risks during surgery (4, 10–13). However, conservative treatment may do little to prevent fatal outcomes in many cases and with new surgical and neuroimaging technological advances, surgical procedures are expected to be more optimistic options for the treatment of PBSH. Therefore, it is meaningful to discuss the controversial but promising surgical management of PBSH in further detail below based on the available evidence.

### Potential Indication of Surgical Interventions

Identifying optimal candidates for surgery is an essential question. Surgical prognostic factors after PBSH conduce to the identification of ideal candidates for PBSH. Through analyses of prognostic factors, Tao et al. concluded that patients with a smaller hematoma (>5 ml and <10 ml), a greater GCS score (>6 and <8), age <65 years, unilateral tegmental hemorrhage, and without extrapontine extension might benefit from surgical treatment (2). Furthermore, based on their experience with five severe cases of surgical treatment, Shrestha et al. proposed their indication for surgery: (1) hemorrhage volume >5 ml (concentrated relatively), (2) GCS score <8 with progressive neural dysfunction, (3) unstable basic vital signs, especially for patients who require mechanical ventilation, (4) location of the hematoma <1 cm from the brainstem surface, and (5) time of hemorrhage <24 h (68).

The indicator by Tao et al. is the equivalent of 2 points in the new PPH score. Four cases in the study by Shrestha et al. scored 2 or 3 points. All the 4 cases survived during their hospital stay and one of them even could go about all daily tasks and walk with minimal help after surgery. According to the findings of Huang et al., a score of 4 points in the new PPH score is the contraindication for both surgery and medical treatments (3). Also, Huang et al. suggested sparing medical resources for patients with a score of 4 points (3). Notably, prompt evacuation of hematoma remains contraindicated in the absence of all brainstem reflexes (68). To sum up, we made the assumption that PBSH patients with a score of 2–3 points in the new PPH score might benefit from surgical management. However, because surgery is not recommended for PBSH based on the current evidence, the assumption requires further verification and should be treated with caution.

As a result of the entirely different anatomical features, blood supply system and the possible distinct cell reactions to hemorrhages (69, 70), the findings and experience of the timing of surgery for supratentorial ICH could not be applied to PBSH directly. Pathological changes observed in animal experiments show that brain edema and arterial necrosis generally appear 6 h after PBSH onset (71). Therefore, in theory, surgery carried out in the super early phase (within a 6 h time window) seems to be the best choice. According to a study by Lan et al., patients with PBSH in the early operative group (≤ 6 h) had a better neurologic recovery than those in the late operative group (>6 h), and this difference was statistically significant (*P* = 0.02) (72). However, based on their experience with 52 cases of surgical treatment, Chen et al. proposed that 12–48 h after ictus may be the optimal surgical timing for PBSH (73).

Overall, as with supratentorial ICH, the exact indicators and the optimal surgical timing for PBSH remain controversial and undetermined (74).

### Anatomical Considerations for Surgery

The ideal surgical approaches often depend on the location and size of the hematoma. The two-point rule by Brown et al. (namely, one point at the center of the hematoma and the other at the point on the brainstem surface to which the hematoma is closest) is frequently used as a means to enter a brainstem hematoma while minimizing disruption of the normal structure (75). However, with the widening knowledge of the anatomy of the brainstem, safe entry zones are considered to have an advantage over the simple two-point rule (76). Various safe entry zones and surgical approaches for the brainstem have been designed to reduce, as much as possible, damage to any eloquent or essential structures. Yang et al. identified 21 different safe entry zones according to the existing literature and endowed each of them with an evidence level (Table 3) (76). Endoscopic endonasal transclival approach (EETA) is a useful approach for endoscopic hematoma removal to provide adequate exposure of the ventral brainstem structure (77). The routine surgical approaches for microsurgery in different brainstem divisions are shown in Table 3 (68).

**Table 3:** Safe entry zones into the brainstem and common surgical approaches (68, 76).

**Brainstem division**	**Safe entry zones**			**Surgical approaches**
	**Case report** **(case number ≤5)**	**Limited evidence** **(5 < case number <25)**	**Credible evidence** **(case number ≥25)**	
Midbrain	Intercollicular region Inferior brachial triangle Interpeduncular fossa zone	Lateral mesencephalic sulcus Anterior mesencephalic zone	Supracollicular/infracollicular zones	Occipital transtentorial approach Subtemporal tentorial approach
Pons	Superior fovea zone Median sulcus zone Area acustica zone Floccular peduncle	Supratrigeminal zone Peritrigeminal zone Lateral pontine zone	Suprafacial zone Infrafacial zone	Suboccipital midline approach Subtemporal tentorial approach Suboccipital retrosigmoid approach
Medulla Oblongata	Posterior intermediate sulcus Posterior lateral sulcus zone Anterolateral sulcus zone	Lateral medullary zone Olivary zone	Posterior median sulcus	Suboccipital midline approach Far lateral approach

### Surgical Options

**Figure 1:**
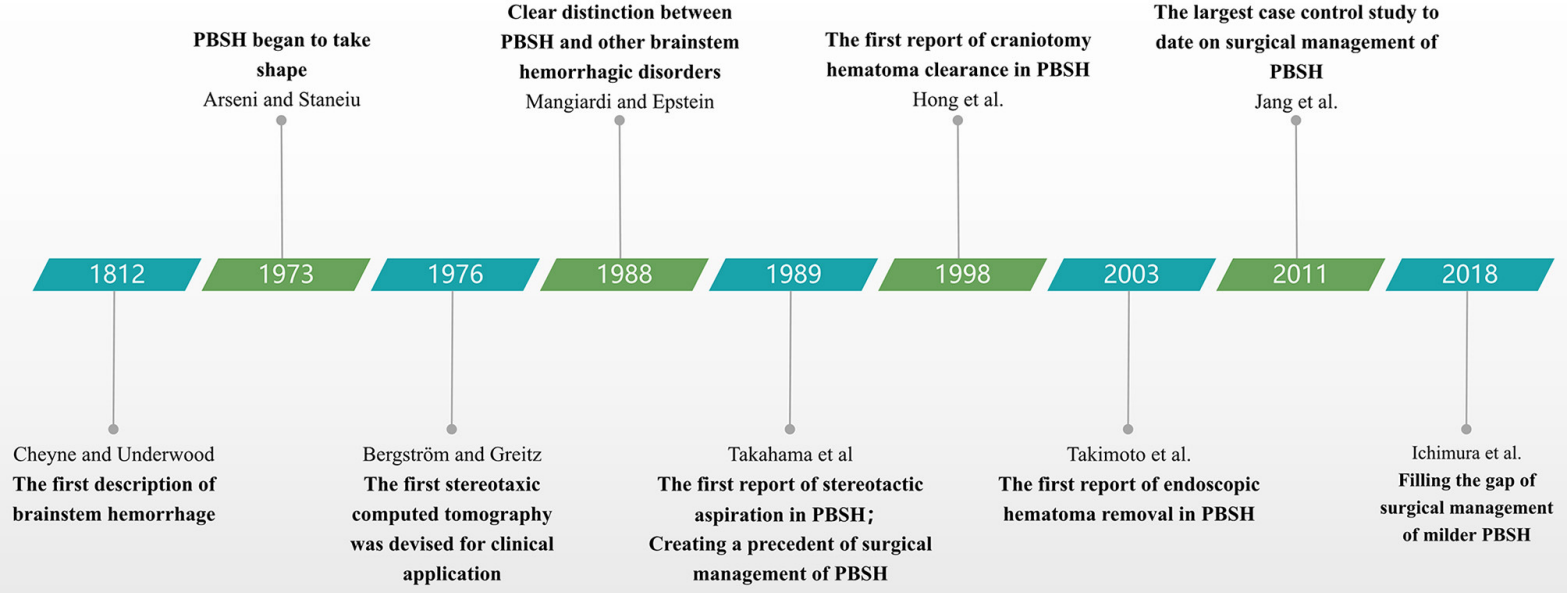
Timeline diagram depicting major milestones for surgical management of PBSH. PBSH, primary brainstem hemorrhage.

Patients with PBSH should be given proper type of surgical options based on the concrete states. Craniotomy is a classic surgical procedure used for PBSH, with advantage of definite hemostasis effect; stereotactic hematoma puncture and drainage is particularly useful for patients who are reluctant to accept craniectomy or are old and feeble; endoscopic hematoma removal could provide adequate exposure of the ventral brainstem lesion when used in a special approach; EVD could be used in emergency medical treatment especially in primary hospitals. We discuss these four main surgical options below and summarize their advantages and disadvantages in Table 4. Major milestones for research on the surgical management of PBSH are presented in Figure 1.

**Table 4:** Comparison of advantages and disadvantages among the four surgical options of PBSH.

	**Craniotomy**	**Stereotactic hematoma puncture and drainage**	**Endoscopic hematoma removal**	**External ventricular drainage**
Advantage	Definite hemostasis effect; concurrent decompression could be performed	Short operation time and easy operation; minimal invasiveness; particularly useful for old and feeble patients 3D-printed navigation (78, 79): high individualized; local anesthesia	Concurrent surgical management of hydrocephalus could be performed (80). EETA: improved direct visualization; adequate exposure of the ventral brainstem structure; minimal brain or neurovascular retraction; a natural surgical corridor with sufficient illumination (77, 81)	A rescue surgical procedure for PBSH in primary hospitals; dynamically monitoring and managing intracranial pressure after the surgery (82)
Disadvantages	Extensive surgical trauma and the possibility of an aggravation of the condition	Special stereotactic equipment The accuracy of 3D print-assisted puncture is slightly lower than that of conventional stereotactic technology (78)	Cerebrospinal fluid leak (83); possible thermal injury to nearby tissues (84); lack of enough experience and high-quality evidence to support EETA in PBSH; a longer learning curve for doctors (77, 84)	A weak therapeutic effect in patients without IVH or hydrocephalus

*CSF, cerebrospinal fluid; EETA, endoscopic endonasal transclival approach; IVH, Intraventricular hemorrhage; PBSH, primary brainstem hemorrhage*.

**1) Craniotomy**

Since suboccipital craniectomy was first used for brainstem hematoma clearance by Hong et al. (85), craniotomy has become one of the most important surgical treatments for PBSH. Lan et al. conducted a case-control study including 286 patients with severe PBSH (GCS ≤ 8), and 46 patients underwent craniotomy under microscope for hematoma clearance (72). Compared with the conservative group, the surgical group had a lower mortality rate (30.4% vs. 70.45%) and a higher good recovery rate (13.1% vs. 5.9%) at the expense of a higher rate of a vegetative state (4.3% vs. 2.5%), severe disability (32.6% vs. 13.3%), and moderate disability (19.6% vs. 7.9%) (72). Ichimura et al. reported the surgical results of five patients with relatively mild PBSH (patients without low initial consciousness and bilateral pupil dilation) (86). All of them had ameliorations in consciousness, motor performance, and mRS grades after surgery (86). Moreover, the authors suggested that the half-sitting position could greatly lower the risk of injury to normal tissue in surgical treatment of brainstem lesions (86). With growing knowledge of safe entry zones and continuing advances in microsurgical techniques (87), satisfactory results could be obtained in a minimally invasive way. Empirical evidence from 52 patients with PBSH indicated that minimally invasive microsurgery for hematoma clearance was very rapid, effective, and safe and was especially suitable for patients with hemorrhage volume <10 ml (73).

**2) Stereotactic hematoma puncture and drainage**

Stereotactic hematoma puncture and drainage was the earliest surgery performed to treat PBSH by Takahama et al. (12). This surgical procedure is easy to perform and has many advantages, such as minimally invasive characteristics and a short surgery time. With the use of stereotactic equipment, anticoagulant urokinase, and rt-PA, it is endowed with high precision and a high hematoma clearance rate. A study by Shitamichi et al. of 45 patients with PPH showed that CT-guided stereotaxic aspiration could improve the prognosis, especially for severe cases (88). In another study enrolling 37 PPH patients, Hara et al. found that 72% (13 of 18) of subjects undergoing CT-guided stereotaxic aspiration showed a dramatic improvement, whereas only 42% (8 of 19) of subjects treated conservatively did (11).

The application of three-dimensional (3D) printing technology is achieving great success in various medical fields, including surgical intraoperative navigation (89, 90). Recently, Wang et al. successfully tested the application of a 3D-printed navigation template for puncture drainage in patients with severe brainstem hemorrhage (78). The actual puncture end was located precisely in the hematoma cavity in all cases, and the postoperative outcomes were satisfactory in all 7 included patients (78). 3D print-assisted hematoma puncture and drainage provides a highly promising new modality for the surgical treatment of PBSH and achieves precision medicine in a completely personalized manner.

With the application of various advanced stereotactic techniques, such as the ROSA (Robotized Stereotactic Assistant) device, stereoscopic virtual reality system, and augmented reality interactive neuronavigation, the surgical procedure would be increasingly safe and precise (91–93).

**3) Endoscopic hematoma removal**

Takimoto et al. were the first to evacuate a pontine hemorrhage with the aid of neuroendoscopy, which provided a new method for the surgical treatment of PBSH (80). However, ventrally located brainstem lesions are still surgically challenging due to their inaccessibility through traditional transcranial approaches. With several advancements, the EETA of neuroendoscopy has gradually become a feasible alternative to treat well-selected ventral brainstem lesions with the advantages of direct visualization and less injury (77). Both Essayed et al. and Weiss et al. proposed the potential feasibility and surgical limitations of EETA to remove ventral brainstem lesions based on cadaveric anatomical studies, and the combination of fiber dissection and 7T-MRI neuronavigation may help us to better understand the clear internal anatomical structure of the brainstem to enter the site of the lesion in a safer manner (94, 95). Topczewski et al. conducted a single-center study of 5 patients undergoing endoscopic endonasal surgery and concluded that EETA could provide enough access to the ventral brainstem (83). Adept operative techniques, the assistance of neuronavigation and intraoperative neurophysiological monitoring are critical for achieving better surgical results. Liu et al. reported a successful case of EETA used in the surgical treatment of a man with severe PBSH (77). An immediate improvement was found in his spontaneous respiration, and his GCS score improved significantly from 3 to 11 1 month after surgery (77). However, there are no other reports of EETA for PBSH. Therefore, EETA used in PBSH remains a surgical challenge that requires further verification of feasibility and surgical limitations based on a large sample.

**4) EVD**

Intraventricular hemorrhage occurs as a rupture of a hematoma into the ventricular system in approximately 39.5% of PBSH patients (6), and it is very frequently involved in elevated intracranial pressure and acute obstructive hydrocephalus due to its physical effect and mass effect (96, 97). EVD is conducive to the clearance of intraventricular blood and the normalization of intracranial pressure (82). Currently, EVD has been used extensively to rescue acute obstructive hydrocephalus and prevent the potential risk of brain herniation induced by high intracranial pressure in the setting of PBSH because no special equipment is required (82, 98). Intraventricular thrombolytics are widely used to dissolve the casting of a hematoma, whereas the recent CLEAR III trial failed to prove a significant improvement in functional outcome with irrigation with alteplase in adult intraventricular hemorrhage (5, 99).

### Clinical Registration Research

Due to the low incidence of PBSH (accounting for 6–10% of spontaneous ICH cases), it is difficult to collect large sample size surgical data within a short time (4). Moreover, in consideration of the high risk, various complications, high treatment costs and uncertain efficacy, the current treatment of PBSH is still mainly conservative. As a consequence, almost all of these previous studies were performed retrospectively with a small sample size, and no high-level evidence is available to support surgical management of PBSH to date.

One ongoing clinical trial, entitled Safety and Efficacy of Surgical Treatment in Severe Primary Pontine Hemorrhage Evacuation (STIPE) (URL: https://clinicaltrials.gov. Unique identifier: NCT04647162), is designed to fill this gap. The STIPE trial is sponsored by West China Hospital and is currently recruiting with an estimated enrollment of 345 participants. Furthermore, it is a multicentric, prospective, randomized, controlled, open-label, clinical study with the objective of evaluating the safety and efficacy of surgical treatment in patients with primary severe PPH (defined as GCS <8 and hemorrhage volume ≥5 ml, the equivalent of 2–4 points in the new PPH score). Patients in the experimental group will receive surgical intervention, such as craniotomy, stereotactic hematoma puncture and drainage or endoscopic hematoma removal, and the control group will only receive conservative medical treatment. The primary outcome measures include the mortality rate and intracranial infection rate at 30 days as well as the rebleeding rate within 3 days after the operation. The secondary outcome measures included the mRS and EQ-5D-5 L questionnaire results at 90, 180, and 365 days after surgery. The study started on January 1, 2021, and the estimated completion date is May 2025.

## Conclusions and Expectations

In conclusion, PBSH has a low incidence but high mortality compared to other forms of ICH in which various prognostic factors are involved. Initial level of consciousness and hemorrhage size are the two most important and consistent predictors and present the two variables in the new PPH score. The new PPH score represents the latest developments in scoring systems and patients with a score of 2–3 points might benefit from surgical management. Therefore, the future direction of scoring systems should verify the availability of the new PPHl score for determining the surgical indications. However, conservative treatment still plays a major role in the management of PBSH and surgery is not recommended for PBSH based on the current evidence. PBSH is always excluded from previous surgical intervention trials for spontaneous ICH, such as the MISTIE III trial, the ICES trial, and the STICH I-II trials (100–103). Besides, in consideration of the complex structures and critical functions of the brainstem, more attention should be paid to potential risks during surgery. Because the number of cases is insufficient, there remains a lack of high-level evidence to prove the efficacy and safety of surgical intervention. The ongoing STIPE trial may fill this gap and provide additional evidence for the surgical treatment of PBSH.

From another point, the plight of clinical studies highlights that animal studies of brainstem hemorrhage are also essential to understand pathophysiological mechanism of PBSH and provide some reference to determining surgical timing, exploring surgical approaches and evaluating surgical efficacy. Though rat models of PPH by autologous blood or collagenase infusion have been established successfully (104–106), there is insufficient evidence to determine whether the pathophysiological difference between supratentorial ICH and PBSH exists. Furthermore, scant attention has been given to PBSH based on the perspective of translational stroke research (106). More efforts should be made in the future to explore pathophysiological features of PBSH and for better translational research.

In summary, we advocate the establishment of a worldwide registry and expert cooperative group to update the epidemiological data (incidence, mortality, etc.), re-evaluate prognostic factors, and re-investigate the surgical indication and timing. Prevention of PBSH also cannot be ignored. Recognizing and controlling risk factors actively are recommended to prevent PBSH.

## Author Contributions

DC, YiT, HN, and PZ conceived the review. DC wrote the paper, with major contributions from WW, QD, GW, MX, YuT, and WL. CP and ZT take responsibility for the manuscript as a whole. All authors contributed to the article and approved the submitted version.

## Funding

This work was supported by grant 81901219 and 82071330 from National Natural Science Foundation of China.

## Conflict of Interest

WL is employed by Beijing WanTeFu Medical Apparatus Co., Ltd. The remaining authors declare that the research was conducted in the absence of any commercial or financial relationships that could be construed as a potential conflict of interest.

## Publisher's Note

All claims expressed in this article are solely those of the authors and do not necessarily represent those of their affiliated organizations, or those of the publisher, the editors and the reviewers. Any product that may be evaluated in this article, or claim that may be made by its manufacturer, is not guaranteed or endorsed by the publisher.
